# PEGylating Ag_2_S Semiconductor Nanocrystals for Pharmacokinetics Tracking: Insights from NIR Luminescence Imaging

**DOI:** 10.1021/acsomega.5c03435

**Published:** 2025-06-26

**Authors:** Irene Zabala-Gutierrez, José Lifante, Nuria Fernandez, Gonzalo Villaverde, Daniel Jaque, Juan Pedro Cascales Sandoval, Jorge Rubio-Retama, Erving Ximendes

**Affiliations:** † MatNaBio Research Group, Pharmacy Faculty, Department of Chemistry in Pharmaceutical Sciences, 16734 Universidad Complutense de Madrid, Plaza Ramón y Cajal S/N, Madrid ES 28040, Spain; ‡ Nanomaterials for Bioimaging Group (nanoBIG), Departamento de Física de Materiales, Facultad de Ciencias, Universidad Autónoma de Madrid, Madrid ES 28049, Spain; § Nanomaterials for Bioimaging Group (nanoBIG), Instituto Ramón y Cajal de Investigación Sanitaria (IRYCIS), Hospital Ramón y Cajal, Madrid ES 28049, Spain; ∥ Institute for Advanced Research in Chemical Sciences (IAdChem), 16722 Universidad Autónoma de Madrid, Madrid ES 28049, Spain

## Abstract

Nanotechnology has revolutionized biomedical applications through the development of nanomaterials with tailored properties, particularly in disease diagnosis and treatment. However, challenges remain regarding the pharmacokinetics of nanomaterials, which influence their biodistribution, targeting efficiency, clearance, and potential toxicity. Near-infrared (NIR) imaging has emerged as a promising tool to study the in vivo behavior of nanomaterials, offering noninvasive, real-time analysis of drug delivery and nanocarrier distribution. Despite its potential, the lack of robust analytical models for precise biodistribution and excretion measurements limits its clinical translation. This study investigates the biodistribution and pharmacokinetics of fluorescent Ag_2_S nanoparticles (NPs) with varying surface charges and capping agent size. These NPs, emitting light at ∼1200 nm in the NIR-II biological window, allow real-time tracking of their distribution. We explored the effects of polyethylene glycol functionalization with different molecular weights on NP behavior. A novel analytical model was developed to assess pharmacokinetic parameters and the influence of surface chemistry on protein–NP interactions. In vitro experiments confirmed that protein binding alters the surface charge and colloidal properties of NPs, which impacts their pharmacokinetics. This work advances our understanding of how nanoparticle surface modifications affect their in vivo performance and interactions with biological systems.

## Graphical abstract

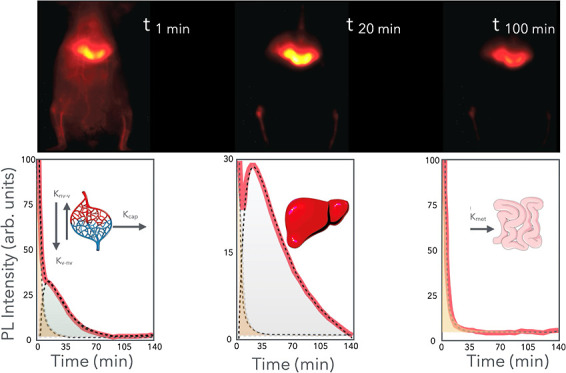

## 1 Introduction

The emerging field of nanomedicine has rapidly developed a rich array of nanomaterials for biomedical applications, including polymers, liposomes, proteins, or inorganic nanoparticles (NPs). These nanomaterials are ultimately administered to living organisms to achieve in vivo fluorescent imaging in preclinical pathological models.^1^ These biological probes can be designed for diagnosis, therapy, or combination of both approaches (theragnosis).

Despite notable advancements in nanomedicine technologies over the past few decades, the clinical translation of nanoformulations is frequently hindered.^2,3^ In this regard, understanding the influence that the physicochemical properties of these nanomaterials exert on their pharmacokinetics and tissue biodistribution is crucial to tailoring their in vivo behavior depending on the application of interest. As a result, nanomaterials could be rationally designed, in terms of, e.g., composition, hydrodynamic diameter, and charge, to improve therapeutic efficacy and minimize toxic effects.^4^

Beyond this, it has been suggested that the development of effective nanomedicines should adopt a disease-driven approach, shifting away from the traditional formulation-driven model that focuses primarily on the engineering of drug delivery systems. This paradigm shift requires a comprehensive understanding of the complex interactions between biological systems and technological innovations, including how disease pathophysiology influences nanomedicine distribution, accumulation, retention, and therapeutic efficacy. Moreover, it highlights the importance of correlating the properties of the nanoformulations with their in vivo behavior in both preclinical animal models and human patients.^5,6^

Numerous techniques are available to evaluate NP biodistribution, including histology, electron microscopy, liquid scintillation counting, indirect drug concentration measurement, optical imaging, computed tomography (CT), magnetic resonance imaging (MRI), positron emission tomography (PET), or single-photon emission-computed tomography (SPECT). Each method offers unique advantages and limitations with varying capacities for real-time imaging, whole-organ visualization, and cellular-level accumulation assessment. In practice, however, NP biodistribution is commonly assessed using radiolabeled nanomaterials, which are typically analyzed post-mortem by measuring radioactivity in different organs and tissues. Unfortunately, this approach necessitates a large number of animal models to construct a biodistribution time course (chronogram) to obtain the NP concentration in each organ at different administration times. Furthermore, the combination of PET/CT or SPECT/CT can provide 3D spatial resolution, offering a clearer picture of the nanomaterial’s location inside the organism. While effective, this methodology is expensive and time-consuming and requires strict safety protocols due to the use of ionizing radiation.^7,8^

In this context, fluorescence imaging within the Near-Infrared Region (NIR, 750 nm–1700 nm) comes into play as an alternative powerful technique for real-time pharmacokinetics tracking of nanomaterials in vivo,^9^ with high sensitivity and spatial resolution, safety, and low cost due to its experimental simplicity.^10^ Real-time experiments could accelerate pharmacokinetic studies at the preclinical level, requiring far fewer animals to perform a time-course behavior profile of nanomaterials within the body.

Ag_2_S nanoparticles have emerged as excellent biocompatible candidates as NIR fluorescence probes, whose excitation (800 nm, NIR-I) and emission (1200 nm, NIR-II) are within the so-called optical biological windows. Imaging within these windows, light scattering, tissue absorption, and tissue autofluorescence are reduced to a minimum, providing improved penetration depth and imaging resolution.^11,12^ Importantly, their relatively high fluorescence efficiency enables the acquisition of whole-body images with low injection doses.^13,14^ These features, along with their chemical versatility, allow them to be incorporated into or functionalized with desired materials as promising nanoplatforms for biodistribution analysis.

Ag_2_S NPs have been widely used for more than 10 years, exploiting their multifunctional properties in vivo, not only as deep-tissue fluorescence imaging probes^15^ but also for photothermal therapy,^16^ photoacoustic imaging,^17^ or nanothermometry.^18^ However, the scarce research about the pharmacokinetic behavior of these nanomaterials^19,20^ reflects the need to obtain straight and accessible methods to track their performance inside the body.

Following the outlined motivation, differently charged Ag_2_S NPs were produced, anchored with poly­(ethylene glycol) (PEG) molecules of different molecular weights (MWs). The aim of this work was to track the differently functionalized NPs in vivo through NIR fluorescence imaging and relate these profiles to their potential as imaging or drug carrier agents. To achieve this, we first evaluated in vitro the influence that different functionalizations exert on the physicochemical and optical properties of the NPs and their consequent formation of the biological protein corona, which has been previously related to a decrease of targeting capabilities and cellular uptake.^6,21^ This potential prediction of the physiological behavior of Ag_2_S NPs in vivo was finally tested by real-time fluorescence tracking in CD1 mice. A four-compartment pharmacokinetic model was developed to obtain pharmacokinetic parameters from the NIR-II emission of the Ag_2_S NPs, key to clarifying how surface chemistry governs nanomaterial distribution in biological environments. The results presented here can provide valuable guidance for the design and optimization of Ag_2_S-based nanomaterials for targeted drug delivery and other biomedical applications.

## 2 Experimental Section

### 2.1 Chemicals

Silver nitrate (AgNO_3_, 99.9%), sodium diethyldithiocarbamate (NaDDTC, ACS reagent grade), oleylamine (OLA, 70%), 1-dodecanethiol (DDT, ≤98%), 11-mercaptoundecanoic acid (MUA, 95%), *N*-(3-(dimethylamino)­propyl)-*N*′-ethylcarbodiimide hydrochloride (EDC, 99%), *N*-hydroxysulfosuccinimide sodium salt (Sulfo-NHS, 98%), methoxypolyethylene glycol amine (PEG-NH_2_, MW = 2000 and 5000 g mol^–1^), and HS-PEG-NH_2_ (MW = 2000 and 3500 g mol^–1^) were purchased from Sigma-Aldrich. Heterofunctional amine PEG thiol (HS-PEG-NH_2_, MW = 600 g mol^–1^) was acquired from NANOCS. Heterofunctional methoxy PEG thiol (HS-PEG-OMe) with different molecular weights (MW = 750, 2000, 5000 g mol^–1^) and heterofunctional methoxy PEG amine (PEG-NH_2_, MW = 800 g mol^–1^) were acquired from RAPP Polymere. All of the materials were used without further purification. Chloroform (CHCl_3_, 99.6%), ethanol absolute pure (99.8%), hexane (99%), and diethyl ether (99.5%) were purchased from PanReac AppliChem.

### 2.2 Physicochemical and Optical Characterization of Ag_2_S NPs

Transmission electron microscopy (TEM) studies were carried out using a Talos F200X microscope operated at 80 kV. Cryo-TEM analyses were performed in a 300 kV JEOL CryoARM. Dynamic light scattering (DLS) and ζ-potential determinations were carried out using a Malvern Zetasizer Nano-ZS instrument. ζ-Potential measurements were performed in Milli-Q water by adding 10 μL of a nanoparticle suspension (0.5 mg/mL) to 1 mL of Milli-Q water. Nanoparticles that had been previously incubated in FBS medium were centrifuged and redispersed in Milli-Q water prior to ζ-potential analysis. For DLS measurements, NPs were dispersed in PBS. However, NPs that had been incubated with FBS were first centrifuged and redispersed in Milli-Q water before DLS analysis. The emission spectra upon illuminating the samples with an 800 nm CW laser were collected with a Bentham ISR300 instrument equipped with an InGaAs detector. Luminescence decay curves were obtained by exciting the colloidal suspensions of NPs with an OPO oscillator (Lotis) tuned to 800 nm, which provides 8 ns pulses at a repetition rate of 10 Hz. Fluorescence intensity was detected with a Peltier cooled photomultiplier tube with enhanced sensitivity in NIR-II (Hamamatsu R5509-73). The contribution of scattered laser radiation was removed using two band-pass filters (FEL850 from Thorlabs) and a high-brightness monochromator (Shamrock 320 from Andor). The time evolution of the fluorescence signal was finally recorded and averaged by a digital oscilloscope (LeCroy WaveRunner 6000).

### 2.3 Cytotoxicity Study of Ag_2_S NPs

Cell viability experiments were performed in the HeLa (human cervix adenocarcinoma) cell line through the alamarBlue (resazurin) assay. HeLa cells were maintained in Dulbecco’s Modified Eagle’s Medium (DMEM) supplemented with 10% fetal bovine serum, 2 mM glutamine, 40 μg mL^–1^ gentamicin, 100 U mL^–1^ penicillin, and 100 μg mL^–1^ streptomycin. Cells were seeded in 24-well plates at a density of 20,000 cells in 0.5 mL per well and cultured in 5% CO_2_ at 37 °C for 24 h. Then, negatively charged, noncharged, and positively charged Ag_2_S NPs were added and incubated for 24 h at different concentrations (0, 5, 10, 25, 50, and 100 μg mL^–1^) and in triplicate. After that time, the cell viability was determined. For this purpose, 0.5 mL of 10% alamarBlue in DMEM was added to each well and incubated for 1 h at 37 °C. Finally, cell culture supernatants were transferred to a plate without cells. The plate was read in an automated TECAN plate reader at an excitation wavelength of 560 nm and an emission wavelength of 590 nm. Corrected emissions were transformed to a cell viability percentage (%) by the following equation:

$$cell\;viability\;\%={emission}_{sample}/{emission}_{control}\times 100$$

where emission_control_ is the emission intensity of cell plates with 0 mg mL^–1^ of Ag_2_S NPs.

### 2.4 In Vivo Experimental Procedures

In vivo experiments were approved by the regional authority for animal experimentation of the Comunidad de Madrid and were conducted in agreement with the Universidad Autónoma de Madrid (UAM) Ethics Committee, in compliance with European Union directives 63/2010UE and Spanish regulation RD 53/2013. For this study, a total of 16 CD1 female mice (8–14 weeks old, weighing 25–39 g) bred at the animal facility at UAM were used. The mice were anesthetized prior to the imaging experiments in an induction chamber with a continuous flow of 4% isoflurane (Forane, AbbVie Spain, S.L.U.) in 100% oxygen until loss of righting reflex was confirmed and breathing rhythm was significantly slowed. Anesthesia was maintained throughout the experiments by means of facemask inhalation of 1.5% isoflurane and the core body temperature was maintained at 36 ± 1 °C, as measured with a rectal probe, using a heating pad. Freshly dissected organs were obtained after animal euthanasia through beheading after 5 min of isoflurane overdose (5%, 2 L/O_2_ min) to ensure total loss of consciousness and pain sensation.

### 2.5 In Vivo NIR-II Hyperspectral Imaging

NIR-II images of in vivo anaesthetized animals and ex vivo freshly dissected organs were obtained by using a homemade NIR-II system. A fiber-coupled diode laser operating at 800 nm was used as the excitation source (LIMO30-F200-DL808). The illumination intensity was controlled via adjustment of the diode current. An anesthetized mouse was placed on a homemade temperature-controlled plate operating at 36 °C. The NIR-II fluorescence image was acquired with a Peltier cooled InGaAs camera (CRED-II). The InGaAs detector was cooled to −10 °C. Two long-pass filters (FEL850 from Thorlabs) were used to remove the background signal generated by the scattered laser radiation (see schematic representation in Figure S3).

### 2.6 Synthesis of Ag_2_S-PEG NPs

The synthesis of the Ag_2_S-PEG NPs was carried out in different steps, as explained in the following sections.

#### 2.6.1 Synthesis of Ag_2_S NPs

Ag_2_S NPs were synthesized following the thermal decomposition method of a AgDDTC precursor, as previously described.^14^ Briefly, the AgDDTC single-source precursor was prepared by reacting 4.25 g of AgNO_3_ (25 mmol) with 5.63 g of NaDDTC (25 mmol), each of them predissolved in 200 mL of Milli-Q water. The addition of the NaDDTC solution to the AgNO_3_ solution resulted in a yellow precipitate (AgDDTC), consecutively filtered under vacuum, and dried at 60 °C using a vacuum evaporator. After that, 25 mg of AgDDTC (0.1 mmol) was typically added into a two-neck round-bottom flask at room temperature, which already contained 2.5 mL of DDT and 2.5 mL of OLA. The reagent mixture was first submitted to vacuum for 10 min to remove air and then filled with N_2_. Thereupon, it was heated to 180 °C under moderate magnetic stirring at a 20 °C min^–1^ heating rate. The reaction was kept for 1 h and subsequently cooled down naturally. The as-synthesized NPs were collected by addition of ethanol and centrifuged at 10,000*g* for 10 min, repeating the washing process twice. The final product was dispersed in 10 mL of CHCl_3_ cooled down to 4 °C and sonicated with the aim of obtaining highly efficient NPs, as already reported.^14^

#### 2.6.2 PEGylation of Ag_2_S NPs

Ag_2_S NPs in CHCl_3_ were transferred to water through PEGylation for their pharmacokinetic tracking in mice. Three different series of PEGylated Ag_2_S NPs were produced in order to obtain negatively charged, noncharged, and positively charged Ag_2_S NPs.

##### 2.6.2.1 Negatively Charged Ag_2_S NP Series (Ag_2_S/MUA/PEG)

Ag_2_S NPs were first treated with MUA for their exchange reaction with hydrophobic ligands. More concretely, 20 mg of MUA was added to 1 mL of dispersion containing Ag_2_S NPs at 1 mg mL^–1^ in CHCl_3_. The mixture was then sonicated in an ultrasonic bath for 10 min reaching the loss of colloidal stability and precipitation at the bottom of the flask. After that, the precipitated NPs were collected and dispersed in 1 mL of water. Carboxylic groups of the MUA molecules provided good colloidal stability in water.

Subsequently, Ag_2_S/MUA NPs were covered with PEG-NH_2_ (MW = 5000 g mol^–1^) via EDC/NHS coupling. For that, 0.5 mg of EDC and 0.7 mg of sulfo-NHS were dissolved in 1 mL of water containing 1 mg of the NPs and 1 mg of PEG-NH_2_. The mixture was gently stirred for 2 h at room temperature, and after that, the NPs were collected by centrifugation using Amicon centrifugal filters (MWCO = 50 kDa) at 9600*g* for 10 min. This process was repeated three times, and the resulting Ag_2_S/MUA/PEG_5000_ NPs were finally dispersed in 1 mL of water and stored at 4 °C.

The procedure was identical for the production of Ag_2_S/MUA/PEG_2000_ and Ag_2_S/MUA/PEG_800_, excepting the PEGylation with PEG-NH_2_ (MW = 2000 g mol^–1^) and PEG-NH_2_ (MW = 800 g mol^–1^), respectively.

##### 2.6.2.2 Noncharged Ag_2_S NP Series (Ag_2_S/PEG-MeO)

Ag_2_S NPs were first sonicated for 5 min in CHCl_3_ at a concentration of 1 mg mL^–1^ using a Branson Sonifier 250, setting the minimum output control (20 W) in a pulsed mode of 0.1 s of sonication per second. The sonicated NPs were transferred from CHCl_3_ to water through their functionalization with HS-PEG-MeO of three different molecular weights (MW = 750, 2000, and 5000 g mol^–1^). Specifically, 5 mg of each PEG was added to 1 mg of NPs dispersed in 1 mL of CHCl_3_. The ligand-exchange reaction was facilitated under vigorous stirring and was maintained for 20 min. Then, 2 mL of hexane was added to destabilize the PEGylated NPs, followed by precipitation via centrifugation (10 s, 1000*g*). The NPs were redispersed in 500 μL of ethanol and 500 μL of water. Ethanol was finally removed by evaporation. The addition of water and evaporation of ethanol was repeated twice to ensure the complete removal of ethanol. PEGylated Ag_2_S NPs were finally dispersed in 1 mL of water and stored at 4 °C for future use.

##### 2.6.2.3 Positively Charged Ag_2_S NP Series (Ag_2_S/PEG-NH_2_)

The method for the production of positively charged Ag_2_S NPs was similar to the one for noncharged NPs, with two protocol variations. The first, sonicated NPs were functionalized with HS-PEG-NH_2_ of three different molecular weights (MW = 600, 2000, and 3500 g mol^–1^). And the second, PEGylated Ag_2_S NPs were destabilized and washed by adding diethyl ether.

## 3 Results and Discussion

The thermal decomposition reaction in organic media yields as a result highly monodisperse Ag_2_S NPs in chloroform with a mean diameter of 8.6 ± 1.3 nm, as seen in Figure 1A,B. These NPs exhibit an intense photoluminescence emission at 1220 nm upon excitation with an 800 nm light, which is featured by a long average photoluminescence lifetime, τ = 3.4 μs (Figure 1C,D). Subsequently, Ag_2_S NPs dispersed in chloroform were transferred to water by functionalizing them with PEG molecules of different molecular weights, finally producing nine samples of PEGylated Ag_2_S NPs with different charges and hydrodynamic diameters (detailed in Figure 1M, see upper panel). PEGylation was the surface-coating selected because it is the most widely used strategy for biomaterials, providing good stability and high biocompatibility.^22^ Negatively charged PEGylated NPs (Ag_2_S/MUA/PEG) were transferred from chloroform to water by the ligand-exchange reaction, resulting in MUA-functionalized Ag_2_S NPs, with 10.1 nm of the hydrodynamic diameter and −49.4 V of the ζ-potential. Three different PEG amines (MW = 800, 2000, and 5000 g mol^–1^) were employed for their coupling reaction with carboxylic residues of Ag_2_S/MUA NPs. The successful PEGylation was proven by the change in ζ-potentials, as shown in Figure 1L. The shorter the PEG length, the more neutral the negative charge of MUA-functionalized NPs. Noncharged and positively charged PEGylated NPs (Ag_2_S/PEG-MeO and Ag_2_S/PEG-NH_2_, respectively) were transferred to water through functionalization of thiolated PEG-MeO and PEG-NH_2_, respectively. The ζ-potential of the resulted NPs is detailed in Figure 1L. Negatively charged, noncharged, and positively charged Ag_2_S NPs resulted in colloidally stable samples in water, as depicted in Figure 1E–G. Upon surface functionalization, all samples exhibited an intense photoluminescence emission at 1220 nm upon excitation at 800 nm, as observed in Figure 1H.

**Figure 1:**
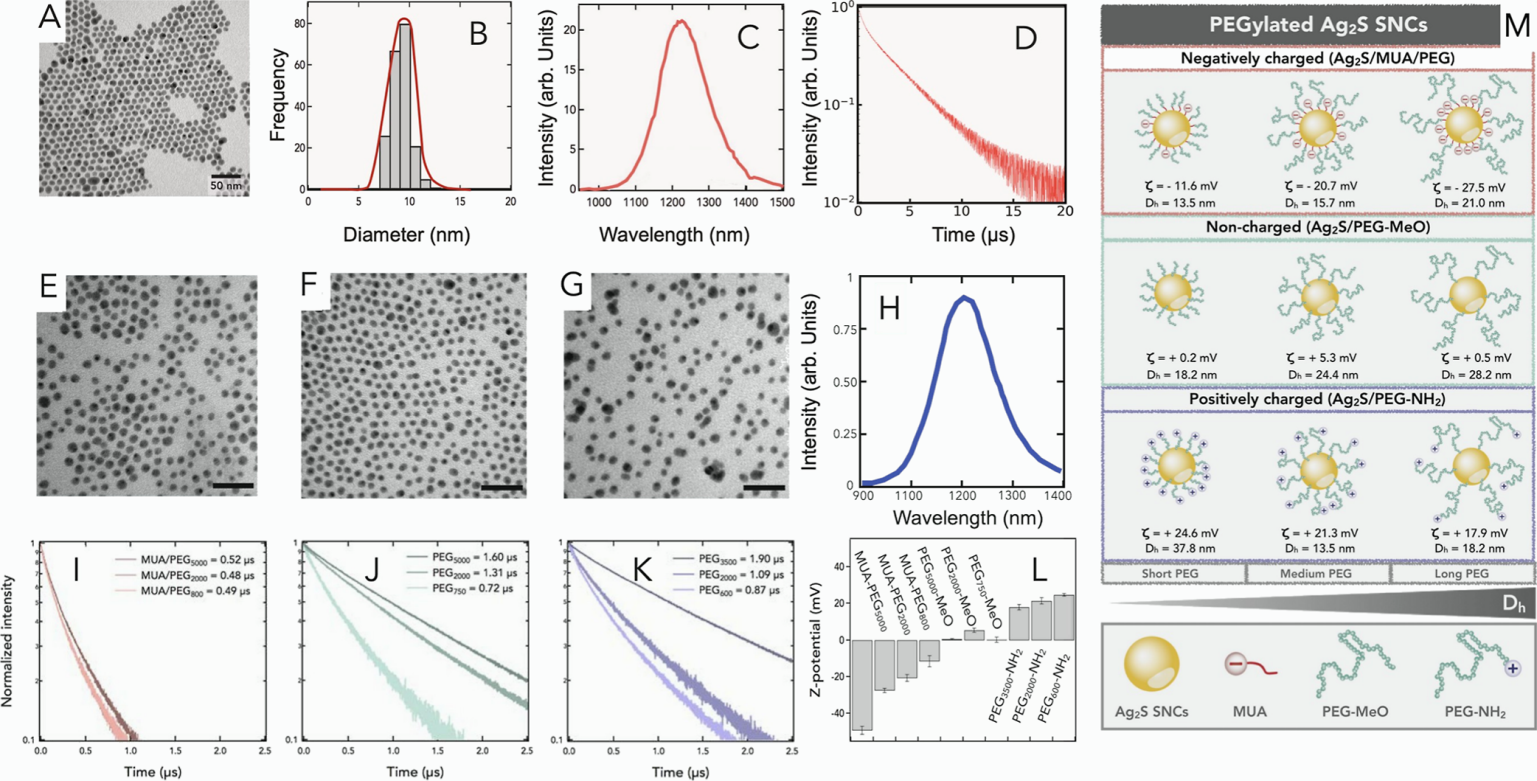
(A) TEM image of the as-synthesized Ag_2_S NPs in chloroform. (B) Size distribution of the NPs obtained from TEM. (C) Photoluminescence spectrum and (D) photoluminescence decay curve of Ag_2_S NPs in chloroform under 800 nm excitation light. TEM images of (E) negatively charged (MUA/PEG_2000_), (F) noncharged (PEG_2000_-MeO), and (G) positively charged (PEG_2000_-NH_2_) Ag_2_S NPs in water. Scale bars: 50 nm. (H) Photoluminescence spectrum of PEGylated Ag_2_S NPs in water. Photoluminescence decay curves of (I) negatively charged (Ag_2_S/MUA/PEG), (J) noncharged (Ag_2_S/PEG-MeO), and (K) positively charged (Ag_2_S/PEG-NH_2_) NPs in water. Labels indicate the average lifetime values calculated from each sample. (L) Comparison of ζ-potential values for PEGylated Ag_2_S NPs and MUA-functionalized NPs in water. Error bars are the standard deviation of three different measurements. (M) Upper panel, schematic representation of negatively charged, noncharged, and positively charged PEGylated Ag_2_S NP series. The *Z*-potential (ζ) and hydrodynamic diameter (*D*_h_) are detailed for each type of NPs. The lower panel represents a schematic illustration of the Ag_2_S NPs functionalized with thiol-terminated PEG molecules of different molecular weights, showing the decrease in grafting density when using longer PEG molecules. The latter entails less incorporation of quenching surface thiols to the NPs, explaining the enhancement of the optical properties of noncharged and positively charged PEGylated Ag_2_S NPs functionalized with the longest PEG.

In a previous work,^14^ the extreme surface sensitivity of the as-synthesized Ag_2_S NPs to functionalization was pointed out. For that reason, we evaluated the optical properties of the different samples. Photoluminescence decay curves were measured, highlighting the influence that the molecules attached to the surface have on the lifetime of Ag_2_S NPs (see Figure 1I–K). Negatively charged series have shown the shortest decay times, keeping comparable lifetime values regardless of the length of PEG used (Figure 1I).

On the other hand, noncharged and positively charged NPs presented longer decay times than negatively charged samples and increasing lifetime values when using longer PEGs (Figure 1J,K). This could be explained in terms of the grafting density of the functionalized molecules on the surfaces of the NPs. As previously investigated,^14^ optimized Ag_2_S NPs proved to be sensitive to thiol-functionalized molecules acting as nonradiative hole traps. For negatively charged samples, it should be first taken into account that MUA molecules and not PEG molecules are the ones interacting with the surface of the NPs. Small MUA molecules would impose lower steric hindrance and thus higher grafting density than PEG molecules, incorporating a higher amount of thiol-related surface traps and consequently decreasing the lifetime values of the negatively charged series. However, when employing thiol-functionalized PEGs in noncharged and positively charged series, a higher PEG molecular weight would result in less incorporation of thiol-related surface traps, leading to longer decay times and PEG-length-dependent lifetime values (see schematic representation in Figure 1M, lower panel).

Hydrodynamic diameters (*D*_h_) of the different PEGylated NPs in water are depicted and summarized in Figure S1, showing a correlation between the length of the PEG molecules selected and the final NPs *D*_h_. However, Ag_2_S NPs functionalized with PEG_600_-NH_2_ displayed the highest *D*_h_ (37.8 nm) of all dispersions, despite using the shortest PEG. The partial aggregation of this sample could be related to a less efficient functionalization of single NPs due to the lower stability of PEG_600_-NH_2_ in chloroform, in which the PEGylation reaction was carried out. In any case, the hydrodynamic diameter always remained below 40 nm, ensuring similar colloidal properties.

Differently charged Ag_2_S NPs have been studied in vitro in terms of their protein corona formation, which consists of the agglomeration of proteins on the surface of the nanomaterials when they are embedded in biological fluids, that has been related to their corresponding pharmacokinetics in vivo.^6,21^

Thus, protein corona formation was promoted by incubating the Ag_2_S NPs in fetal bovine serum (FBS).^23^ More concretely, each type of NPs was incubated in 0.5 mL of FBS (50% in PBS) at a similar concentration (50 mg mL^–1^) for 1 h at 25 °C. The incubated NPs were carefully centrifuged (21,000*g*, 10 °C, 30 min) and redispersed in PBS to remove the unbounded proteins. The washing process was repeated three times.^24^ Similar experiments of each type of NPs in PBS were performed as control samples. Dynamic light scattering (DLS) and ζ-potential measurements were performed to characterize the protein corona formed around the differently charged Ag_2_S NPs, see Figure 2A–F. Hydrodynamic diameters increased after the incubation in FBS for noncharged (Figure 2B) and positively charged NPs (Figure 2C), while the negatively charged series did not evidence a significant change in size (Figure 2A), indicating a minor protein corona formation.^20^ In addition to that, all Ag_2_S samples were found to present a ζ-potential value of ∼−15 mV after FBS incubation (Figure 2D–F),^24^ being consistent with the charge of the albumin protein, which is the most abundant component in the protein corona.^25,26^ Cryo-TEM observations of the Ag_2_S NPs confirmed the greater agglomeration of NPs after protein corona formation of positively charged Ag_2_S NPs, while negatively charged NPs were shown isolated in the FBS dispersion (Figure 2H–M).

**Figure 2:**
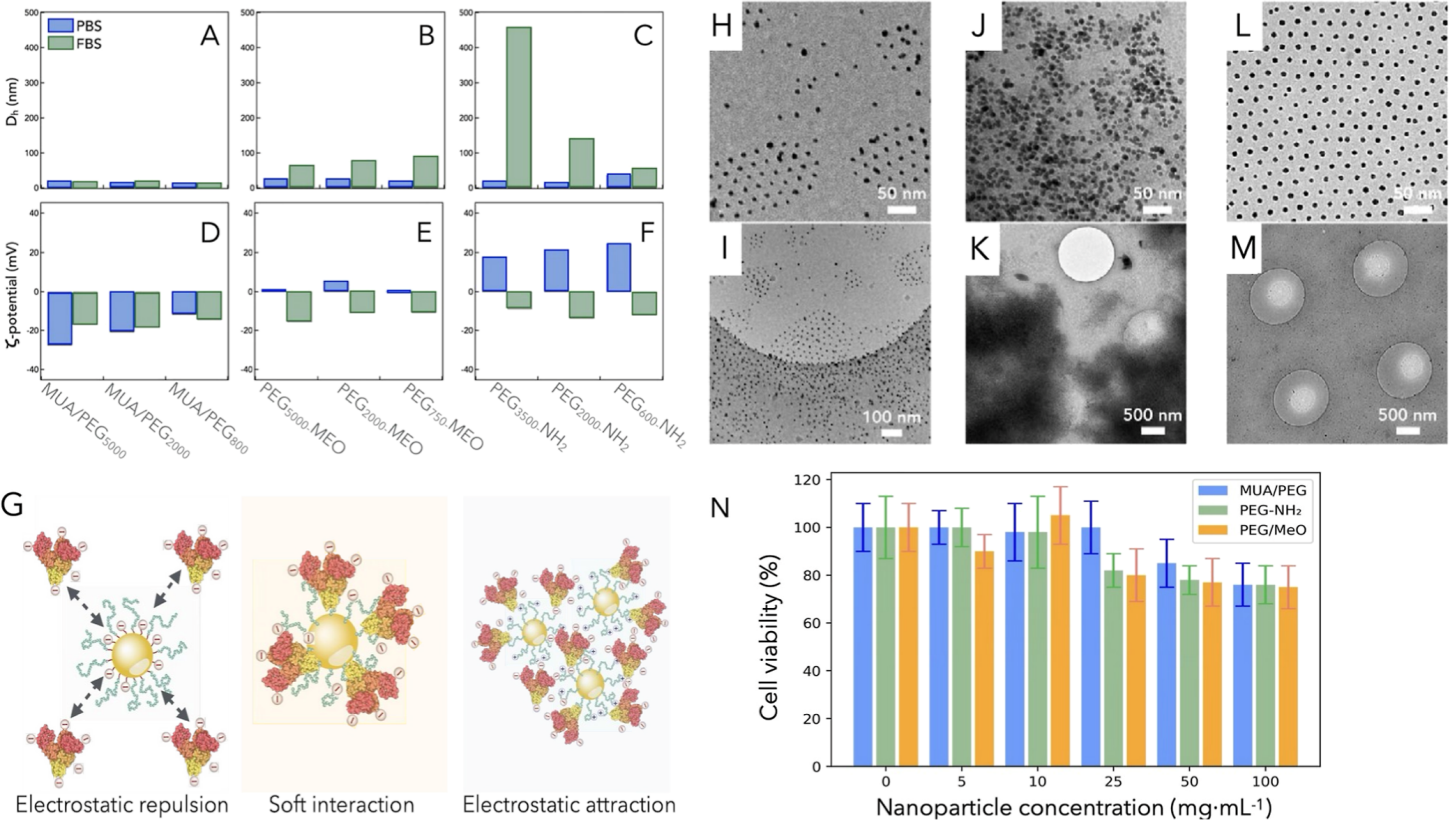
In vitro protein corona formation of PEGylated Ag_2_S NPs. (A–C) Hydrodynamic diameter (*D*_h_) and (D–F) ζ-potential changes of PEGylated Ag_2_S NPs before and after incubation in FBS. (G) Illustration of the interaction between differently charged Ag_2_S NPs with albumin from biological fluids. Cryo-TEM pictures at different magnifications of (H,I) positively charged Ag_2_S/PEG_2000_-NH_2_ in PBS, (J,K) positively charged Ag_2_S/PEG_2000_-NH_2_ in FBS, and (L,M) negatively charged Ag_2_S/MUA/PEG_2000_ in FBS for the protein corona formation evaluation in FBS. (N) Cell viability of HeLa cells after 24 h of incubation in the presence of negatively, noncharged, and positively charged PEGylated Ag_2_S NPs. Mean values and standard deviation error of each group (*n* = 3) are represented.

Going into more detail, ζ-potential and DLS measurements provided valuable insights into the interactions between polymers and NPs. These interactions are likely governed by electrostatic forces between the positively charged groups on the NPs and the negatively charged residues on albumin molecules. This electrostatic attraction leads to the formation of a negatively charged complex, characterized by a reversal of charge due to the binding of albumin to the NPs. In contrast, negatively charged NPs would experience electrostatic repulsion with albumin, resulting in a weaker or negligible interaction. This reduced interaction is reflected in the maintenance of both the hydrodynamic diameter and the charge of the NPs, as albumin does not significantly alter their surface characteristics. Figure 2G visually summarizes these charge-mediated interactions between albumin and NPs, highlighting the influence of the surface charge on the degree of interaction.

As previously mentioned, protein corona formation has been related to the circulation and clearance of NPs from the body. Therefore, protein corona in vitro experiments were carried out as a potential predictor of the Ag_2_S NP physiological behavior in vivo.^27^ The former experiments resulted in a minor protein corona formation for negatively charged PEGylated Ag_2_S NPs, which would mean a prolongation of their in vivo circulation time, in agreement with previous reports.^20,21,28^ As a conclusion, charge-dependent behavior of PEGylated Ag_2_S NPs in FBS would allow forecasting their different response if administered in vivo, in terms of circulation time and biodistribution. Therefore, Ag_2_S functionalization should be carefully selected according to the requirements of the bioapplication of interest.

Although Ag_2_S NPs have already demonstrated only moderate cellular toxicity in dose ranges of mg mL^–1^, tolerated by in vitro and in vivo systems,^13^ changes in surface functionalization might also affect toxicity. For this reason, prior to any evaluation of the pharmacokinetics of PEGylated Ag_2_S NPs in vivo, in vitro cell viability tests were conducted as a function of the NP surface charge. The results showed negligible cytotoxic effects on cells under the experimental conditions tested, exhibiting cell viabilities higher than 80% at NP concentrations below 100 mg mL^–1^ (Figure 2N).

To evaluate the pharmacokinetics of the different PEGylated Ag_2_S NPs, a series of in vivo experiments in mice were carried out. Retro-orbital injections targeting the systemic circulation were performed to evaluate changes in NIR-II fluorescence biodistribution patterns (CD1 female mice; 2 *n* = 2 per group; 16 in total). 150 μL of each dispersion was injected at a selected concentration of 0.8 mg mL^–1^ of Ag_2_S NPs in PBS. That would correspond to a total injected dose of ≈ 4 mg kg^–1^, below injection doses previously employed for NIR-II in vivo fluorescence imaging with Ag_2_S NPs.^29,30^ Mice were shaven and placed in a supine position to facilitate tracking of the luminescence generated by the NPs that circulated throughout the entire organism. The Ag_2_S NPs were excited with an 808 nm wavelength and fluorescent signals within the 1050–1600 nm range were collected with an infrared camera (ZephIR 1.7) (Figure 3A and Section S3). The imaging process commenced immediately after injection and continued for a period of 180 min. A detailed description of the administration, animal preparation, and illumination can be found in the Experimental Section.

**Figure 3:**
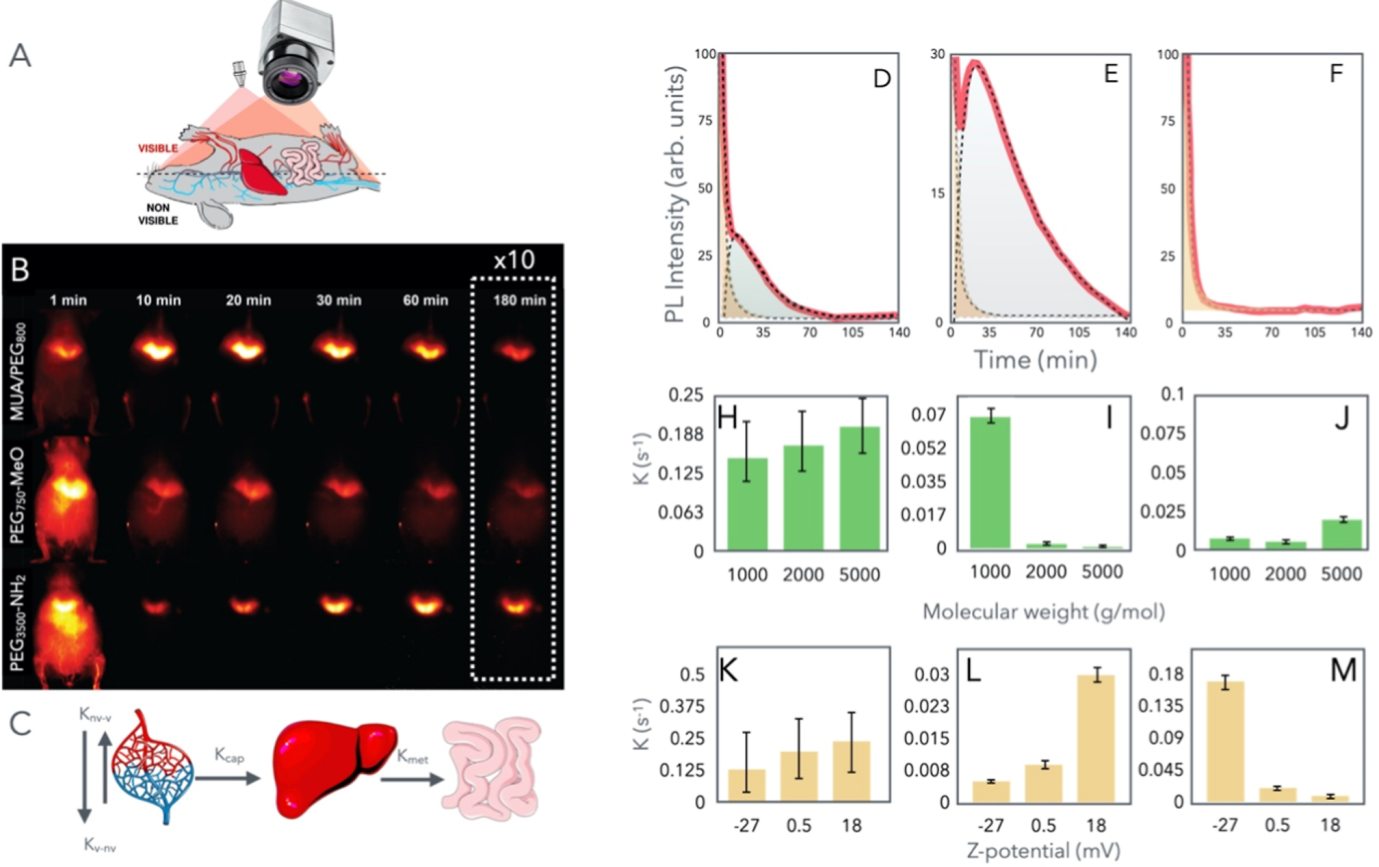
(A) Schematic representation of the animal position used to record the NP signal distribution. (B) NIR-II images of negatively charged (first row), noncharged (second row), and positively charged (third row) PEGylated Ag_2_S NPs recorded in mice at different times from NP administration. The contrast of images at 180 min was modified for ease of visualization. (C) Schematic representation of the pharmacokinetic model used in the data analysis, showing the four compartments (visible vasculature, nonvisible vasculature, liver, and gastrointestinal tract) and the kinetic constants obtained. (D–F) Integrated NIR-II signal intensity as a function of time obtained from the whole animal model, the liver region, and the rest of the animal model, respectively. Representation of the distribution rate (H), liver capture (I), and metabolic rate (J) obtained as a function of the PEG molecular weight. Representation of the distribution rate (K), liver capture (L), and metabolic rate (M) obtained as a function of the NP *Z*-potential.

Figure 3B presents representative luminescence images obtained at various time points for the differently charged PEGylated Ag_2_S NPs. Observations revealed a common trend of preferential accumulation of the Ag_2_S NPs in the liver across all series, widely known as the main clearance mechanism for these types of NPs.^20,31^ However, notable differences were observed in the timing of the maximum accumulation.

As a first step to gain a deeper understanding of the underlying mechanisms governing NP accumulation in the liver, Principal Component Analysis (PCA) was applied to the acquired videos. The results (see Section S5) highlight three regions with different dynamics: vasculature, liver, and gastrointestinal (GI) tract. We then developed a simplified particle-exchange numerical model based on the organs that could be observed in vivo under our experimental conditions. Both visible and nonvisible vasculature were considered as the direct supplier of NPs to the liver (Figure 3A), with the liver having the capacity to receive and clear NPs into the GI tract. Guided both by what we observed in the videos captured during the experiments (Figure 3B) and by the PCA results (Figure S4), we proposed a four-compartment model, namely, visible vasculature, nonvisible vasculature, liver, and GI tract (Figure 3C). The video frames were used to calculate the NP emission over time (which is proportional to the NP concentration) in the whole animal model, represented in Figure 3D. We then divided the animal model into two regions: the liver (Figure 3E) and the rest of the animal model (Figure 3F).

We have assumed the following: (i) the signal from the mouse excluding the liver is due to visible vasculature and gastrointestinal contributions; (ii) there is a redistribution of the NPs between visible and nonvisible vasculature compartments; (iii) the signal from the region of interest (ROI) in the liver is due to the accumulation of NPs in the liver and, to a lesser degree, to liver vasculature and circulation above the liver, whose luminescence signal is also captured by the camera. Our model further assumes unidirectional NP uptake from the blood by the liver, followed by NP hepatic clearing dependent on biliary secretions in the GI tract. For simplicity, the kinetics used in the model are all first order.

Therefore, we propose the following set of differential equations which describe the major features observed in the experimental data due to NP kinetics in the system:

1 $$\frac{dC_{v}\left(t\right)}{dt}=-\left(k_{v‐nv}+k_{capt}\right)C_{v}\left(t\right)+k_{nv‐v}C_{nv}\left(t\right)$$

2 $$\frac{dC_{liv}\left(t\right)}{dt}=k_{capt}\left(C_{v}\left(t\right)+C_{nv}\left(t\right)\right)-k_{met}C_{liv}\left(t\right)$$

3 $$\frac{dC_{gi}}{dt}=k_{met}C_{liv}\left(t\right)$$

4 $$\frac{dC_{nv}\left(t\right)}{dt}=-\left(k_{nv‐v}+k_{capt}\right)C_{nv}\left(t\right)+k_{v‐nv}C_{v}\left(t\right)$$

where *C*_v_, *C*_nv_, *C*_liv_, and *C*_gi_ are the Ag_2_S NP concentrations in the visible vasculature, nonvisible vasculature, liver, and GI tract, respectively. The rate constants correspond to the NP redistribution from the visible to nonvisible vasculature and vice versa (*k*_v‑nv_, *k*_nv‑v_), NP uptake from the vasculature by the liver (*k*_capt_), and the elimination of NPs from the liver to the intestine or the “metabolic” rate (*k*_met_).

As each experiment was performed with a different animal, and there are, therefore, variations in imaging conditions between experiments, we model the signals in the liver L and the rest of animal W as

5 $$W\left(t\right)=\alpha C_{v}\left(t\right)+\beta C_{gi}$$

6 $$L\left(t\right)=\gamma C_{v}\left(t\right)+\delta C_{liv}\left(t\right)$$

The functions L and W defined this way give us the needed flexibility to account for imaging differences between experiments, e.g., animal physiology, relative position of the different organs with the camera, amount of tissues between the camera and liver, etc. Because the parameters α, β, γ, and δ already contain information regarding the compartment area and NP concentration (which is unknown), we define the following simple boundary conditions:

7 $$C_{v}\left(0\right)=1-\chi$$

8 $$C_{liv}\left(0\right)=0$$

9 $$C_{gi}\left(0\right)=0$$

10 $$C_{nv}\left(0\right)=\chi$$

where χ is the fraction of NPs which are in the nonvisible vasculature compartment at *t* = 0. Lastly, we needed to account for the fact that the image acquisition did not begin immediately after injection as the mice had to be positioned within the imaging system. This delay, *t*_offset_, was around 2 min, and it has been incorporated into the model as a fixed constant (*t*_offset_ = 2 min).

This fit of the model to the experimental data is carried out using GNU Octave^32^ and consists of applying nonlinear regression with a function which makes use of an ordinary differential equation solver. From each experiment, we obtain the following fitting parameters: *k*_v‑nv_, *k*_nv‑v_, *k*_capt_, *k*_met_, χ, α, β, γ, δ. All values obtained for the fitting parameters are shown in Table S1.

Pharmacokinetic data of Ag_2_S NPs, obtained from the four-compartment pharmacokinetic model, seems to indicate that, after the administration, the NPs were promptly cleared from the bloodstream and accumulated preferentially in the liver (see Figure 3D). Nevertheless, the steep decay at the very beginning of the experiment must be understood considering that once the NPs enter the animal model, they are distributed along the entire circulating system, with a large fraction of NPs reaching areas localized deep inside the tissues and below the imaging plane, and are therefore considered to be in the nonvisible vasculature. As a result, many of the administered NPs reduce their contribution in the integrated emission intensity due to the absorption of light by the tissues.

However, 10 min after the administration, the presence of a shoulder can be observed, with its maximum 20–30 min after the injection (Figure 3D). This is the result of the accumulation of the NPs around the hepatic region that, due to its superficial localization and the position used to analyze the animal, is more susceptible to being observed. In fact, when we analyze the integrated intensity only in the hepatic region (Figure 3E), this accumulation is clearly observed. The graph shows an initial decay of the integrated intensity followed by a sudden rise that reaches a maximum around 30 min after the administration, finally decreasing monotonically for the remainder of the experiment. The first decay is related to the vascular system localized in the hepatic region, which exhibits the same profile as the one observed in the circulating system shown in Figure 3F. Thus, if we assume that the initial decay is due to the circulating system, we can extract the pharmacokinetics of the liver, and from which it is possible to infer two clearance rates: (i) the capture rate (*K*_capt_) corresponding to the speed at which the liver clears NPs from the circulating system, being responsible for the intensity increment peaking at ∼30 min and (ii) the “metabolic” rate (*K*_met_), related to the capacity of the liver to metabolize the NPs and to transform them into a non-emitting material, which corresponds to the decrease of the signal intensity observed at longer times (shadowed in grey in Figure 3D,E).

Under these assumptions, we have analyzed the effect that the surface chemistry exerts on the different kinetic constant rates. In this vein, when we analyze the effect of the molecular weight on the distribution constant rate (*K*_v‑nv_), we can observe that, essentially, the molecular weight does not significantly modify the obtained values (Figure 3H). By contrast, when we analyze this effect on the capture constant rate (*K*_capt_), we can observe that this is inversely proportional to the molecular weight (Figure 3I). That means that the capacity of the liver to clear NPs from the circulating system is hampered when the NPs are covered with a PEG of high molecular weights. That would impose a steric hindrance as well as reduced interaction capacity with the proteins of the hepatocytes.^21^ This result agrees with those observed when noncharged, PEGylated NPs were incubated with FBS, and this highlights that NPs covered with a PEG of higher molecular weights formed a smaller protein corona (small *D*_h_). Finally, concerning the *K*_met_, we have not observed a significant variation in the results obtained, what could indicate that once the NPs are captured by the liver, the degradation pathways are not affected by the studied hydrodynamic sizes (Figure 3J).

More information can be obtained when we analyze these kinetic constant rates as a function of the NP surface charge. For instance, the analysis of the distribution constant as a function of the superficial charge gives as a result a small increment of the *K*_v‑nv_ when the charge becomes more positive (Figure 3K). This could be related to a lower venous return due to the increase in hydrodynamic size experienced by positively charged NPs. This could hinder the passage of NPs from the arterial to the venous circulation through the capillaries.^33^

Likewise, the analysis of *K*_capt_ as a function of the surface charge gives as a result an increment of *K*_capt_, for positively charged NPs (Figure 3L). This effect can be related to the higher capacity to interact with proteins through electrostatic interaction. This is supported by the result shown in Figure 2A–C, i.e., the *D*_h_ of the NPs in FBS increases when the surface charge becomes more positive. Thus, the strong interaction between proteins and NPs would render larger protein corona formation and a faster clearance of these NPs from the bloodstream.

Another interesting effect observed when the charge of the NPs becomes positive is a significant reduction of the *K*_met_ (Figure 3M). In fact, when we observe Figure 3B, the emission intensity obtained from the hepatic region at longer times, we can clearly see that the intensity of the signal in positively charged NPs is significantly higher when compared with noncharged and negatively charged NPs. Interestingly, this is also supported by the PCA results (Figure S4), where the vasculature and GI tract both show similar dynamics (vasculature and GI tract are not as differentiated) for negative NPs, with the numerical model yielding rate constants *K*_v‑nv_ and *K*_met_ which are of the same order of magnitude. For neutral and positive NPs, both the PCA and numerical model reveal markedly different circulation and clearing rates. This result could indicate that the presence of positive charge hinders the degradation of NPs once they enter the liver. These results clearly evidenced that the liver uptake and retention was more pronounced for positively charged NPs, in agreement with previous reports.^34^

To finalize the pharmacokinetic study of PEGylated Ag_2_S NPs in mice, we performed a biodistribution analysis by measuring the NIR-II luminescence signals from different organs ex vivo 180 min after the administration. As seen in Figure 4A, luminescence pictures show different biodistribution patterns, depending on the superficial charge of the PEGylated NPs. When the integrated luminescence intensity is represented and compared between organs (Figure 4B), the first conclusion to notice is that the liver and spleen, as the main organs involved in the reticuloendothelial system, retained the majority of Ag_2_S NPs, regardless of the NP functionalization.^19^ The gastrointestinal tract, lungs, thymus, heart, and bones also showed partial accumulation of NPs, while the rest of the organs brought much lower luminescence signals 180 min after the NP administration. Interestingly, negatively charged PEGylated Ag_2_S NPs were further retained in lungs, when compared to noncharged and positively charged NPs. The latter has been previously observed and related to the protein corona composition in negatively charged NPs, which facilitates their interaction with lung epithelial cell receptors, being finally internalized.^35^ Regarding the gastrointestinal tract, noncharged PEGylated Ag_2_S NPs were considerably retained.^36^ This could be understood by the fact that non-negative surface charges may interact more favorably with the mucosal surface of the gastrointestinal tract, whose negative charge is primarily attributed to the presence of glycosaminoglycans and sialic acids, facilitating the adhesion and retention of the NPs in the intestinal lining.^37^ Also, the considerable amount of Ag_2_S NPs accumulated within bones (as shown in the sternum luminescence signal), probably evidencing the macrophage uptake of NPs in the bone marrow.^19,20^

**Figure 4:**
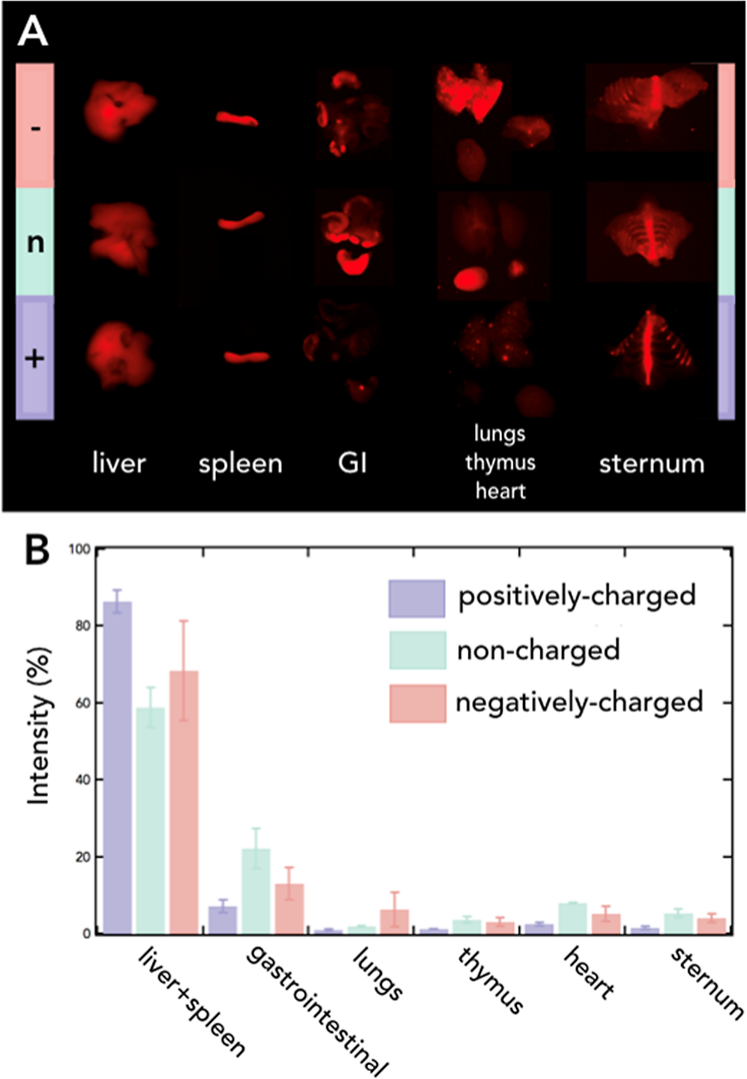
(A) Ex vivo NIR-II intensity pictures from different organs of mice sacrificed 180 min after the negatively charged (first row), noncharged (second row), and positively charged (third row) NP administration. Pictures show the following organs: liver (first column), spleen (second column), gastrointestinal tract (GI, third column), lungs, thymus, heart (fourth column), and sternum (last column). (B) NIR-II intensity percentage (%) for different organs as calculated from ex vivo images in (A).

Consequently, from these in vivo luminescence pharmacokinetics tracking and ex vivo biodistribution study with PEGylated Ag_2_S NPs, we have been able to understand and highlight several key observations: (i) for angiography purposes and pulmonary research, NPs with negative surface charges are preferable, (ii) for investigations concerning the gastrointestinal tract, noncharged NPs are more suitable, and (iii) positively charged NPs should be rationally selected to study hepatic physio-pathologies.

In summary, the results presented here support the conclusion that the biodistribution of nanoparticles is predominantly governed by two critical parameters: the molecular weight (MW) of the poly­(ethylene glycol) (PEG) moieties coating the NPs and their surface charge. Both factors play essential roles in mediating the interactions between NPs and biological components such as serum proteins and cell surface receptors, as denoted by the DLS measurements and the hepatic uptake.

With respect to the PEG molecular weight, we observed that all NPs, irrespective of PEG MW, exhibited comparable kinetic parameters: *k*_nv‑v_ ≈ 0.01 s^–1^ and *k*_v‑nv_ ≈ 0.193 s^–1^. These results suggest that as long as the NPs remain colloidally stable and maintain a sufficiently small hydrodynamic diameter, they can circulate systemically without aggregating or accumulating nonspecifically in tissues. However, a marked increase in the liver uptake rate (*k*_cap_) was detected in NPs coated with low-MW PEG. This observation underscores the importance of steric stabilization. High-MW PEG chains form a denser and more extended protective layer around the NPs, effectively reducing opsonization by serum proteins and subsequent recognition by hepatic phagocytic cells. In contrast, low-MW PEG provides less steric hindrance, leading to increased protein adsorption and faster liver accumulation. Notably, the rate of NP clearance from the liver appears to be largely unaffected by the PEG MW, suggesting that excretion mechanisms are not significantly influenced by the steric profile once hepatic uptake has occurred.

Regarding surface charge, our results indicate that the general biodistribution pattern of NPs remains relatively stable across different surface charges as long as the NPs retain colloidal stability. Nevertheless, we observed a significant increase in the hepatic uptake rate (*k*_cap_) when the NPs possessed a more positive surface charge, with values increasing from 0.007 to 0.03 s^–1^ as the superficial charge of the NPs shifted from negative to positive. This trend is likely driven by enhanced electrostatic interactions between positively charged NPs and negatively charged serum proteins and cell membranes, leading to greater opsonization and recognition by liver macrophages (Kupffer cells). In contrast, negatively charged NPs tend to experience electrostatic repulsion from similarly charged biological components, which reduces the level of nonspecific binding and protein corona formation. As a result, negatively charged NPs show lower liver uptake and slower clearance, whereas positively charged NPs demonstrate increased hepatic accumulation and more rapid clearance.

All of this highlights the crucial role that surface chemistry plays in the NP’s biodistribution, accumulation, and excretion of nanoparticles with similar hydrodynamic diameters. The surface interface of the NPs is the primary region where interactions with biomolecules occur. This suggests that the nature of the inorganic core has a negligible effect as long as the surface-bound organic molecules sufficiently isolate the core from the biological environment.

## 4 Conclusions

In this study, we investigated the impact of surface functionalization on the pharmacokinetics of differently charged PEGylated Ag_2_S NPs. First, we performed an in vitro experiment which consisted in the incubation of Ag_2_S NPs in biological serum, evidencing a minor protein corona formation for negatively charged NPs. These results were correlated with the pharmacokinetics study in vivo, achieved by developing a four-compartment model to obtain pharmacokinetic parameters from the NIR-II emission of Ag_2_S NPs in mice. In accordance with the in vitro experiments, negatively charged NPs exhibited slow clearance from the liver, potentially due to electrostatic repulsion with negatively charged cell membranes. On the other hand, positively charged NPs showed a rapid uptake and prolonged retention by the liver. The conclusions here presented were backed up by the application of principal component analysis to the pharmacokinetics videos, elucidating the essential role of surface charge in differentiating luminescence intensity dynamics. Finally, an ex vivo analysis of the NIR-II luminescence signals of different organs provided comprehensive information on the biodistribution patterns of the differently charged PEGylated Ag_2_S NPs. This work highlights the suitability of negatively charged NPs for angiography, non-negatively charged nanoparticles for gastrointestinal tract studies, and positively charged NPs for hepatic physio-pathological research. The presented findings could pave the venue for future applications of Ag_2_S NPs in fluorescent bioimaging. Overall, our study offers insights that can contribute to the design and optimization of NPs for targeted drug delivery where the choice of surface functionalization directly influences the biodistribution, clearance, and therapeutic efficacy.

## Supplementary Material



## Notes

Additional characterization results, experimental setup, fitting parameters, and principal component analysis (PDF)
