# Supplementary material for: PEGylating Ag2S Semiconductor Nanocrystals for Pharmacokinetics Tracking: Insights from NIR Luminescence Imaging
Source: ACS Omega. 2025 Jun 26;10(26):28351–61. doi: 10.1021/acsomega.5c03435 (PMC12242633; doi:10.1021/acsomega.5c03435)
Supplement: Supplementary file 1 [file ao5c03435_si_001.pdf]

Supporting Information:

PEGylating Ag_2S Semiconductor Nanocrystals for Pharmacokinetics Tracking: Insights from NIR Luminescence Imaging

Irene Zabala-Gutierrez1, José Lifante2, Nuria Fernandez2, Gonzalo Villaverde1, Daniel Jaque 2, Juan Pedro Cascales Sandoval1,*, Jorge Rubio-Retama1,*, Erving Ximendes2,*

1. Universidad Complutense de Madrid, Pharmacy Faculty, Department of Chemistry in Pharmaceutical Sciences, MatNaBio Research Group, Plaza Ramón y Cajal S/N, Madrid, ES 28040

2. Universidad Autonoma de Madrid, Nanomaterials for Bioimaging Group (nanoBIG), Departamento de Física de Materiales, Facultad de Ciencias, Universidad Autónoma de Madrid, Madrid, Spain. Nanomaterials for Bioimaging Group (nanoBIG), Instituto Ramón y Cajal de Investigación Sanitaria (IRYCIS), Hospital Ramón y Cajal, Madrid, Spain. Institute for Advanced Research in Chemical Sciences (IAdChem), Universidad Autónoma de Madrid Madrid, ES 28049

Section S1. Characterization of PEGylated Ag_2S NPs.

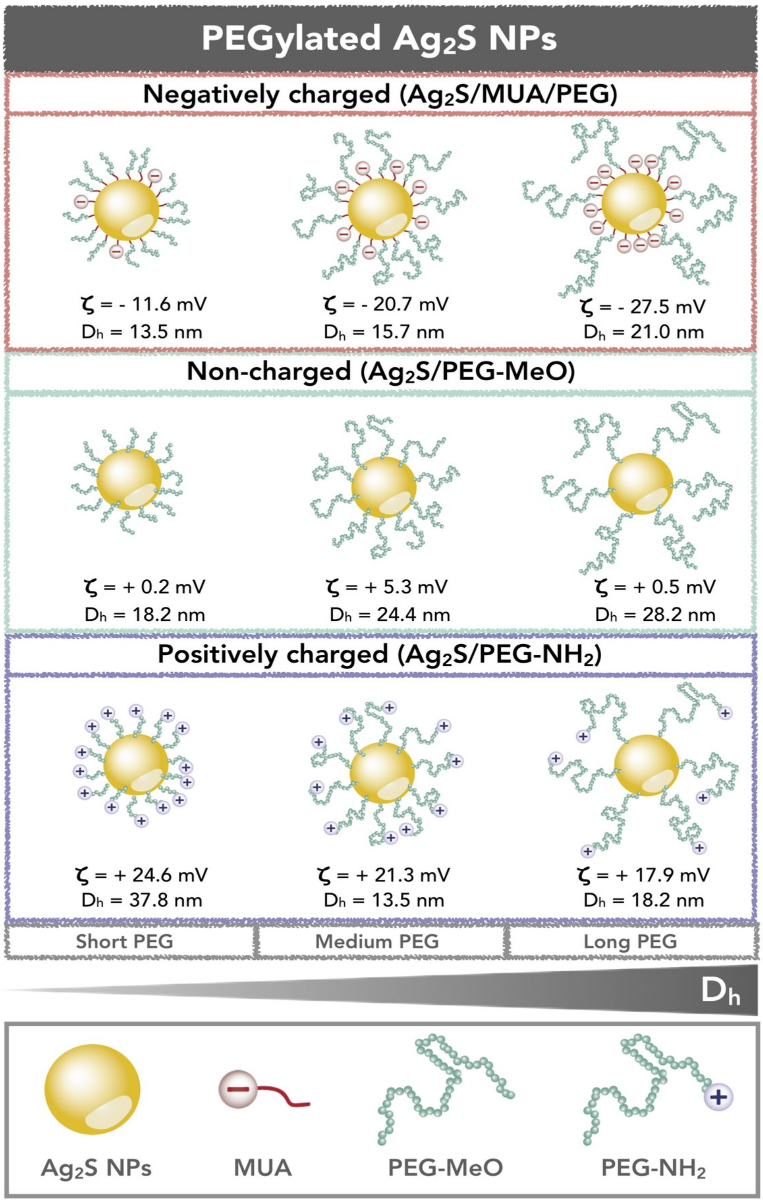

Figure S1. Schematic representation of negatively charged, non-charged and positively charged PEGylated Ag_2S NPs series. Z-potential (ζ) and hydrodynamic diameter (D_h) are detailed for each type of NPs.

Section S2. PEG-length-dependent lifetime values in non-charged and positively charged PEGylated Ag_2S NPs

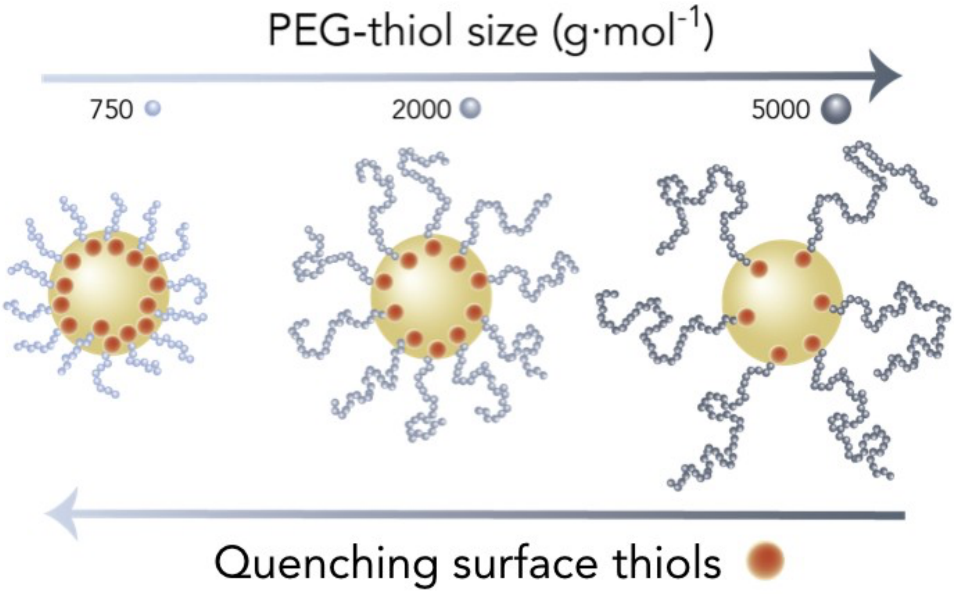

Figure S2. Schematic illustration of the Ag_2S NPs functionalized with thiol-terminated PEG molecules of different molecular weights, showing the decrease in grafting density when using longer PEG molecules. The latter entails less incorporation of quenching surface thiols to the NPs, explaining the enhancement of the optical properties of non-charged and positively charged PEGylated Ag_2S NPs functionalized with the longest PEG.

Section S3. *In vivo* tracking of PEGylated Ag_2S NPs.

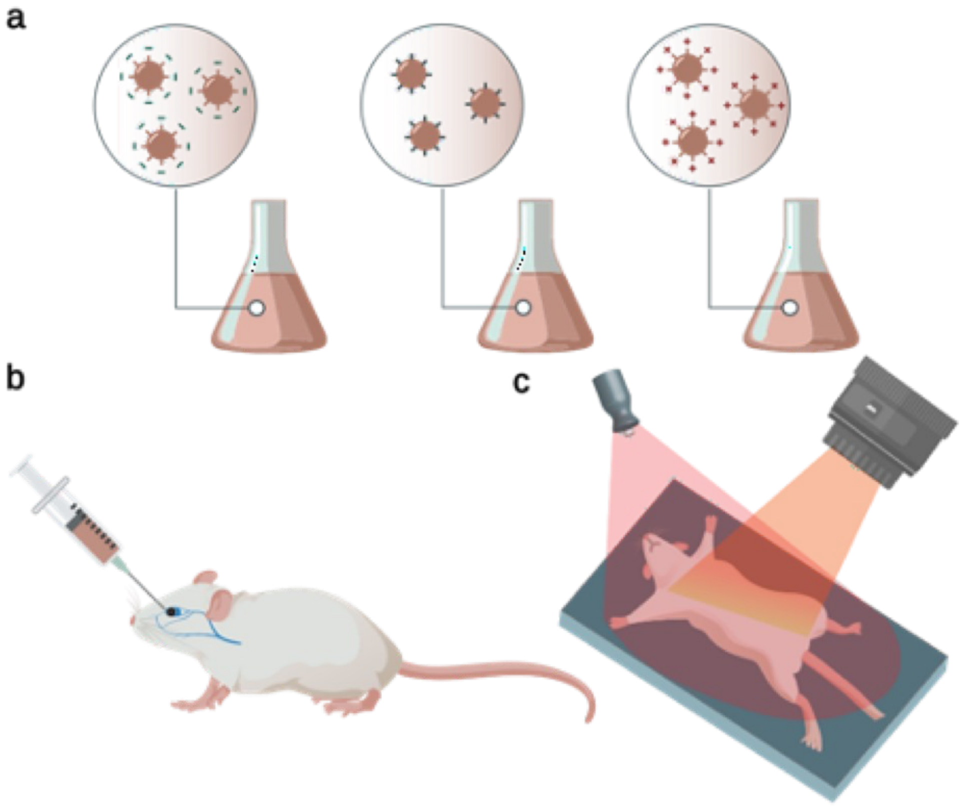

Figure S3. A) Schematic representation of the series of PEGylated Ag_2S NPs with negative (left), neutral (center), and positive (right) surface charges. B) Schematic representation of the administration route. C) Experimental setup utilized for tracking the Ag_2S NPs throughout the body.

Section S4. Fitting parameters of the biodistribution numerical model.

Parameter	PEG_{750}-MeO	PEG_{2000}-MeO	PEG_{5000}-MeO	MUA-PEG_{800}	MUA-PEG_{2000}	MUA-PEG_{5000}	PEG_{600}-NH_2	PEG_{2000}-NH_2	PEG_{3500}-NH_2
k_{v-nv} (min^{-1})	0.153	0.177	0.209	0.095	0.647	0.131	0.027	0.313	0.241
k_{capt} (min^{-1})	0.070	0.003	0.009	0.005	0.025	0.005	0.064	0.105	0.029
k_{met} (min^{-1})	0.008	0.068	0.027	0.158	0.454	0.173	0.008	0.017	0.009
k_{nv-v} (min^{-1})	0.021	0.006	0.007	0.006	0.050	0.008	0	0.075	0.001
χ	0.346	0.545	0.454	0.444	0.076	0.324	0.201	0.086	0
α	1.778	2.387	2.172	1.938	3.689	1.358	1.094	1.681	1.316
β	0.086	0.158	0.065	0.059	0.014	0	0.009	0.020	0.005
γ	0.433	0.605	0.515	0.221	0.342	0.182	0.338	0.299	0.267
δ	0.094	1.554	0.108	2.861	21.045	3.316	0.016	0.234	0.303

Table S1. Values obtained for the parameters of the proposed biodistribution numerical model, resulting from the fitting of the experimental data for all types of NPs.

Section S5. Principal Component analysis (PCA) of the PEGylated Ag_2S NPs pharmacokinetics videos.

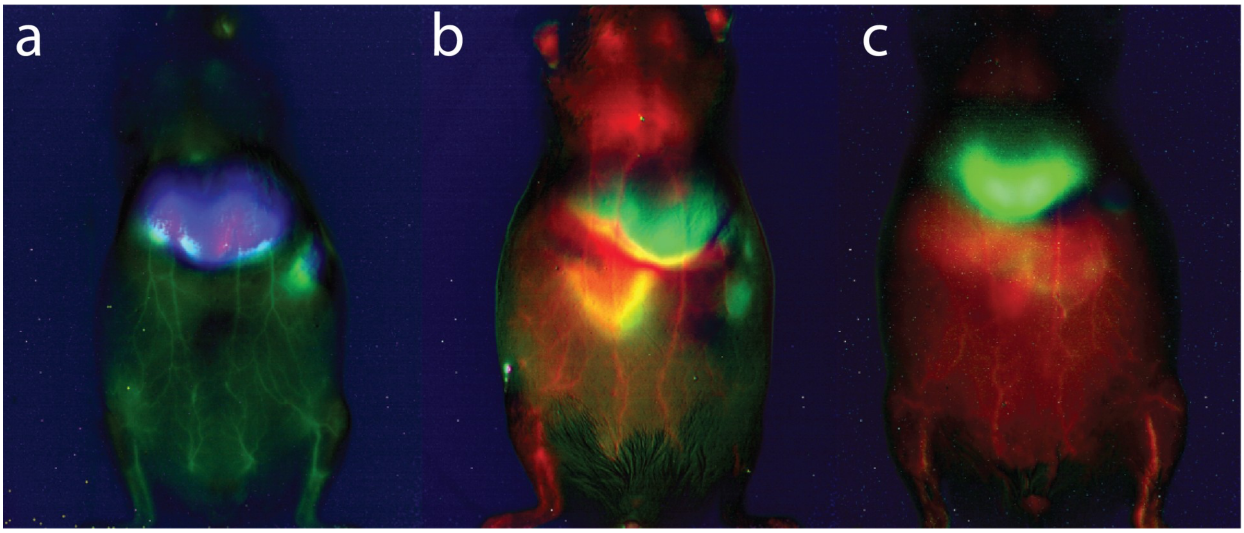

Figure S4. Images acquired after applying Principal PCA for the compression of pharmacokinetics videos of (A) negatively charged (B) non-charged (C) a positively charged PEGylated Ag_2S NPs.

To assess the potential implications of the findings presented in this study, principal component analysis (PCA) was applied to the acquired pharmacokinetics videos. By incorporating the first three principal components into the red, green, and blue (RGB) channels, we generated images as depicted in **Figure S4**. In these images, pixels with similar colors exhibit comparable luminescence intensity dynamics.
